# Association between Apoϵ4 allele and cardiometabolic and social risk factors with cognitive impairment in elderly population from Bogota

**DOI:** 10.1590/1980-57642021dn15-040011

**Published:** 2021

**Authors:** Olga Lucia Pedraza, Isis Camacho, Fabio Alexander Sierra, Rubio-Gómez Cladelis, Ana Maria Salazar, Maria Camila Montalvo, Hector Daniel Morillo, Angela Lozano, Luz Dary Gutiérrez-Castañeda, Lilian Torres-Tobar, Cesar Piñeros

**Affiliations:** 1 Neurosciences Group, Fundacion Universitaria de Ciencias de la Salud – Bogotá, Colombia.; 2 Interdisciplinary Memory Group, Hospital Infantil Universitario de San Jose – Bogotá, Colombia.; 3 Epidemiology and Biostatistics Research Group, Fundacion Universitaria de Ciencias de la Salud – Bogotá, Colombia.; 4 Basic Health Science Group, Fundacion Universitaria de Ciencias de la Salud – Bogotá, Colombia.; 5 Psychology, Cognitive Processes and Education Group, Universidad El Bosque – Bogotá, Colombia.; 6 Medicine Faculty, Fundacion Universitaria de Ciencias de la Salud – Bogotá, Colombia.

**Keywords:** Apoϵ4 allele, mild-cognitive impairment, dementia, MoCA test, educational status, cardiometabolic risk factors., Apoϵ4, comprometimento cognitivo leve, demência, teste MoCA, escolaridade, fatores de risco cardiometabólico

## Abstract

Being an ϵ4 carrier in the Apoϵ gene has been suggested as a modifying factor for the interaction between cardio-metabolic, social risk factors, and the development of cognitive impairment.

**Objective:**

The main objective of this study was to assess the existence of such interaction in a sample of Bogota’s elderly population.

**Methods:**

A cross-sectional study was conducted with 1,263 subjects older than 50 years. Each participant was diagnosed by consensus, after neuropsychological and neuropsychiatric evaluations, under a diagnosis of normal cognition, mild cognitive impairment (MCI) according to Petersen’s criteria, or dementia according to DSM-IV criteria. Apoϵ was typified and an analysis of MoCA test was performed in each group carrying or not ϵ4 allele.

**Results:**

Our study showed that 75% were women with a median age of 68 years (interquartile range 62–74 years) and a median schooling for 6 years (interquartile range 4–12 years). Dementia was related to low education level of ≤5 years OR=11.20 (95%CI 4.99–25.12), high blood pressure (HBP) OR=1.45 (95%CI 1.03–2.05), and age over 70 years OR=7.68 (95%CI 3.49–16.90), independently of being or not an ϵ4 allele carrier. Diabetic subjects with dementia carrying ϵ4 allele showed a tendency to exhibit lower scores on the MoCA test, when compared with noncarriers’ diabetic subjects with dementia.

**Conclusions:**

The presence of ϵ4 allele does not modify the relationship between cognitive impairment and the different cardio-metabolic and social risk factors, except in diabetic subjects ϵ4 carriers with dementia who showed a tendency to exhibit lower scores of the MoCA test, when compared with noncarriers’ diabetic subjects with dementia.

## RESUMO

Ser um portador ϵ4 no gene Apoϵ tem sido sugerido como um fator modificador da interação entre fatores cardiometabólicos, de risco social e o desenvolvimento de comprometimento cognitivo.

**Objetivo:**

O objetivo principal deste trabalho é avaliar a existência de tal interação em uma amostra da população idosa de Bogotá.

**Métodos:**

Um estudo transversal foi realizado com 1.263 indivíduos com mais de 50 anos. Cada participante foi diagnosticado por consenso após avaliações neuropsicológicas e neuropsiquiátricas, sob um diagnóstico de cognição normal, comprometimento cognitivo leve de acordo com os critérios de Petersen ou demência de acordo com os critérios do Manual Diagnóstico e Estatístico de Trastornos Mentais (DSM-IV). Apoϵ4 foi tipificada e uma análise do Montréal Cognitive Assessment Test (teste de MoCA) foi realizada em cada grupo portador ou não do alelo ϵ4.

**Resultados:**

Nosso estudo mostrou que 75% eram mulheres com idade mediana de 68 anos (intervalo interquartil 62 a 74 anos) e escolaridade mediana de seis anos (intervalo interquartil 4 a 12 anos). A demência estava relacionada ao baixo nível de escolaridade ≤5 anos *Odds Ratio* (OR)=11,20 (intervalo de confiança — IC95% 4,99–25,12), pressão alta OR=1,45 (IC95% 1,03–2,05) e idade acima de 70 anos OR=7,68 (IC95% 3,49–16,90), independentemente de ser ou não portador do alelo ϵ4. Indivíduos diabéticos com demência portadores do alelo ϵ4 mostraram tendência de exibir pontuações mais baixas no teste MoCA quando comparados com indivíduos diabéticos com demência não portadores do alelo ϵ4.

**Conclusões:**

A presença do alelo ϵ4 não modifica a relação entre o comprometimento cognitivo e os diferentes fatores de risco cardiometabólico e social, exceto em diabéticos portadores de ϵ4 com demência, que exibiram tendência a apresentar menores escores no teste MoCA quando comparados com indivíduos diabéticos com demência não portadores do alelo ϵ4.

## INTRODUCTION

Dementia has become an epidemic, because of the alarmingly worldwide elderly population increase, but with a modest benefit derived from therapeutic advances.^1^ ^,^ ^2^ ^,^ ^3^ Thus, the strategies to prevent and control risk factors have been outlined as useful therapeutic options in the control and evolution of this pathology.^4^

An association between modifiable and nonmodifiable risk factors has been suggested, which has allowed a better understanding of the intervention as an overriding mechanism of prevention.^5^ The main risk factors described for Alzheimer’s dementia (AD), the most frequent dementia, are age, low schooling, cardiovascular and metabolic diseases (e.g., high blood pressure (HBP) and diabetes), genetic and inherited factors, and some lifestyles.^6^ ^‒^ ^10^

Norton et al. described an increased relative risk (RR) for dementia with the presence of HBP and diabetes between 1.61 and 1.46, respectively,^7^ whereas in other studies, low schooling was related to the appearance of dementia in 1 of 5 subjects.^10^

Meanwhile, Riedel et al.^11^ and Santos et al.^12^ described that carrying the ϵ4 allele in the Apoϵ gene was the main genetic risk factor related to the development of AD. Other studies of clinical–genetic correlation showed that ϵ4 allele carriers have a 10-fold higher risk of late sporadic AD if they are homozygous, or 2–3-fold higher risk if they are heterozygous.^13^ In the last years, some studies have postulated a possible interaction between genetic and cardio-metabolic risk factors, which would contribute to the development of cognitive impairment, considering that the ϵ4 allele of the apolipoprotein E gene is a risk factor common for dementia and cardiovascular disease.^8^

Some authors also observed that the presence of vascular risk factors such as HBP combined with the presence of ϵ4 allele would increase the possibility of presenting cognitive impairment.^14^ ^,^ ^15^ ^,^ ^16^ Zou et al.^17^ and Yamazaki et al.^18^ reported that diabetic subjects and ϵ4 allele carriers had a higher prevalence of AD (7.55%) as compared to diabetic non-ϵ4 carriers (2.3%), and concluded that diabetic ϵ4 carriers’ subjects would have a 3,982 [95%CI 1,418–11,184] greater probability of developing dementia as compared to diabetic ϵ4 noncarriers.^17^

Regarding the presence of social risk factors (age or low schooling), being an ϵ4 carrier was related to the development of cognitive impairment according to the study made by Qian et al.^19^ and Kivipelto et al.^20^

For the above, we aimed to evaluate the association between cognitive impairment, being an ϵ4 carrier and having a social, cardiovascular, or metabolic risk factor in a sample of elderly population from the city of Bogota.

## METHODS

This study is part of the analysis of risk factors made in the study about cognitive impairment in adults from Bogotá, in which 1,263 autonomous adult subjects were invited to participate with a family member. Subjects who attended the invitation received an explanation of the study and those who agreed to participate were cited with their respective family member or companion.^9^

The inclusion criteria of the study were being 50 years of age or older, being autonomous living in community, and resident in Bogota without previous cognitive evaluation. Subjects with neuro-psychiatric illness history, illiterate, blind, deaf, and institutionalized were excluded from the study.

### Procedure

After signing the informed consent, socio-demographic, health, and cardiovascular risk questionnaires were applied to each participant as well as a neuropsychological and neuropsychiatric assessment in two phases and each one was diagnosed by consensus, under a diagnosis of normal cognition, MCI according to Petersen’s criteria, or dementia according to DSM-IV criteria. Subjects with the last two diagnoses were referred to their medical service.^21,^ ^22^

Body mass index (BMI) was calculated using measures of weight and height, and for the genotyping of ApoE, a blood sample was taken, and subsequently DNA extraction was performed.^23^ ^,^ ^24^

### Statistical analysis

Central tendency and dispersion measures were used to describe quantitative variables, and absolute and relative frequencies to describe qualitative variables.

The MoCA-test score was compared among patients with and without risk factors, within each group of patients, ϵ4 carriers and noncarriers using Mann-Whitney U test, given that the distribution of this variable was not normal. Also, when statistical significance was reached, a non-parametric effect size, using Cliff’s Delta, was calculated (<0.147 “negligible,” <0.33 “small,” <0.474 “medium,” otherwise “large.”^25^ A possible relationship between cognitive impairment, risk factors, and being or not ϵ4 allele carrier was explored through a multiple correspondence analysis: the illustrative variable was being or not ϵ4 carrier and the active variables were risk factors and cognitive group.

Subsequently, a regression analysis was applied using the MoCA-test score as the dependent variable and as independent variable the interaction between the APOΕ (ϵ4 carriers *versus* noncarriers) and the risk factors (diabetes, HBP, dyslipidemia, overweight, low schooling, and age over 70 years), adjusting the data obtained for each cognitive group (normal, MCI and dementia) by age. A non-parametric quantile regression was used, considering that MoCA-test score did not follow a normal distribution. The risk factor, low schooling, was analyzed in two ways: as five or less years or as ten or less years of schooling, which correspond in our context to primary or incomplete bachelor, respectively. High schooling were high-school graduates, technicians, and university students. Statistical analysis was performed using the R (libraries FactoMineR and effsize) and Stata 13® programs. Significance was assessed at p<0.05 corrected for multiple comparisons using the Boferroni correction.

### Ethics

This study was approved by the FUCS Human Research Ethics Committee and complies with the requirements of the Helsinki Declaration and Resolution 8430 of 1993 on research with human beings in Colombia.

## RESULTS

For this analysis, 1,214 subjects who had complete data on the risk factors assessed diabetes mellitus (DM), HBP, dyslipidemia, overweight, low schooling, and age over 70 years and APOΕ genotyping were included.

Approximately 75% were women with a median age of 68 years (interquartile range [IQR]) age 62 and 74 years and a median schooling for 6 years (IQR) 4 to 12 years; 43.5% of the participants were cognitively normal, 34.1% had MCI, and 22.4% dementia (Table 1).

**Table 1.** Absolute and relative frequency of sociodemographic characteristics, and vascular and metabolic risk factors according to cognitive status.

Characteristics	Cognition			Total 1,214
	Normal 528 (43.5%)	MCI 414 (34.1%)	Dementia 272 (22.4%)	
Sociodemographic features				
Female gender	384 (72.7)	313 (75.6)	217 (79.8)	914 (75.3)
Age-median (IQR)	64 (60–70)	70 (64–74)	73 (66–79)	68 (62–74)
Age in categories				
50–59 years	123 (23.3)	48 (11.6)	12 (4.4)	183 (15.1)
60–69 years	264 (50)	158 (38.2)	87 (31.9)	509 (41.9)
≥70 years	141 (26.7)	208 (50.2)	173 (63.6)	522 (43.0)
Schooling-median (IQR)	11 (5–17)	5 (3–11)	4 (2–5)	6 (4–12)
Schooling in categories				
0 a 5 years	147 (27.8)	211 (50.9)	217 (79.8)	575 (47.4)
6 a 10years	76 (14.4)	82 (19.8)	30 (11.0)	188 (15.5)
≥11 years	305 (57.8)	121 (29.3)	25 (9.2)	451 (37.2)
Body mass index				
<26	228 (43.2)	177 (42.8)	101 (37.1)	506 (41.7)
26–29	190 (35.9)	136 (32.9)	99 (36.4)	425 (35.0)
≥30	110 (20.8)	101 (24.4)	72 (26.5)	283 (23.3)
Vascular and metabolic risk factors				
DM	63 (11.9)	59 (14.3)	45 (16.5)	167 (13.8)
HBP	212 (40.2)	195 (47.1)	163 (59.9)	570 (46.9)
Overweight	300 (56.8)	237 (57.3)	171 (62.9)	708 (58.3)
Dyslipidemia	167 (31.6)	153 (36.9)	86 (31.6)	406 (33.4)

DM: diabetes mellitus, HBP: high blood pressure; MCI: mild cognitive impairment. Values are expressed as absolute numbers and percentages of the study population in parenthesis.

The frequency of our APOΕ genotype, previously described, was distributed as follows: ϵ3/ϵ3 73.4%, ϵ3/ϵ4 20.2%, ϵ2/ϵ3 4.4%, ϵ4/ϵ4 1.4%, ϵ2/ϵ4 0.4%, and ϵ2/ϵ2 0.08%. A total of 22% of participants were carriers of the ϵ4 allele, being 1.5% with homozygous genotype and 20.6% heterozygous. Allelic frequency was ϵ3 85.5%, ϵ4 11.9%, and ϵ2 2.6%.^26^

### Normal subjects: MoCA-test performance in ϵ4 allele carriers and noncarriers and their different risk factors

In normal subjects, both carriers and noncarriers, a lower MoCA-test score was found in those who had equal or less than five years of schooling compared to subjects with more than 5 years of schooling, carriers: median 24.5 [IQR: 21.5– 27] *versus* 26 [IQR: 25–28], p=0.010, Cliff’s delta=–0.30 (small); noncarriers: median 24 [IQR: 20–26] versus 27 [IQR: 25–28], p=0.000, Cliff’s delta=-0.45 (medium).

The same was observed in subjects with equal or less than 10 years of schooling compared to those who had 11 or more years of schooling, carriers: median 25 [IQR: 22–28] *versus* 26 [IQR: 25–28], p*=*0,048, Cliff’s delta=0.21 (small); and noncarriers: 25 [IQR: 22–27]) *versus* 27 [IQR: 25–28], p*=*0.000, Cliff’s delta=0.43 (medium).

Likewise, subjects with 70 years of age or older had a lower MoCA-test score compared to younger elderly population, carriers: median 24.5 [IQR: 21.5–27 *versus* 26 [IQR: 25–28], p*=*0.007, Cliff’s delta=0.35 (medium); noncarriers: median 24 [IQR: 20–26]) *versus* 27 [IQR: 25–28], p*=*0.043, Cliff’s delta=0.12 (negligible).

**Figure 1.**
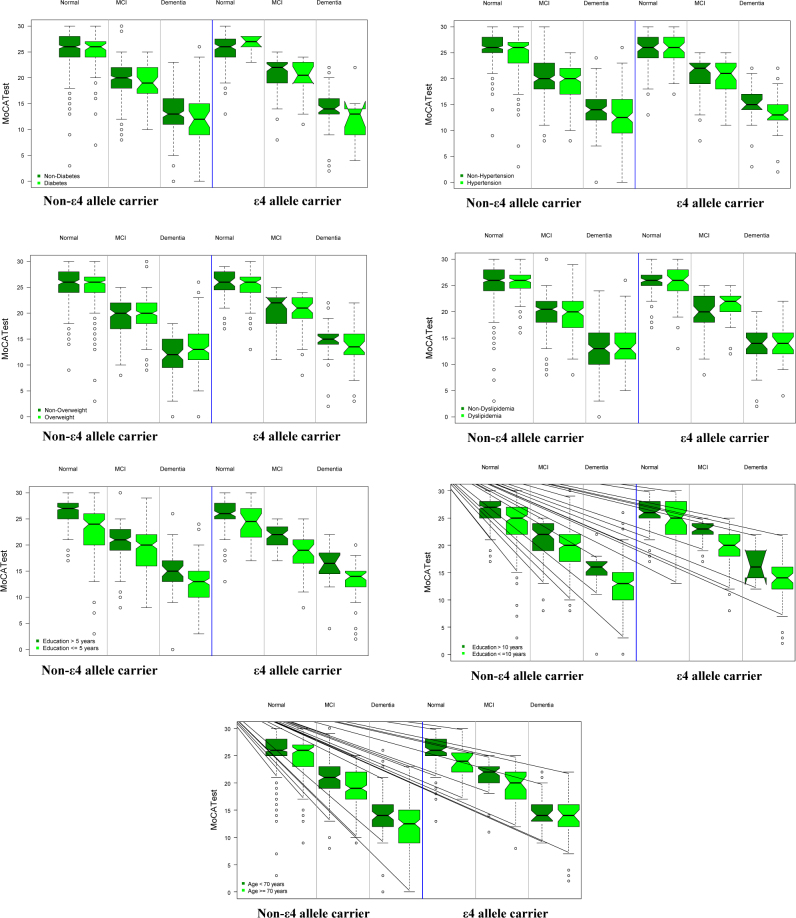
Performance in the Montréal Cognitive Assessment Test according to the carrier status or not of allele ε4, cognitive status and the presence or absence of the risk factor.

There were no differences for ϵ4 allele carriers and noncarriers in the MoCA-test score, between subjects with or without other risk factors (DM, HBP, overweight, or dyslipidemia) (Table 2 and Figure 1).

**Table 2.** Comparison of the Montréal Cognitive Assessment Test, by risk factors, between participants without cognitive impairment (normal), carriers and non-carriers of the allele ε4.

Risk factor		Non-ε4 allele carrier		p-value	ε4 allele carrier		p-value
		Without the factor	With the factor		Without the factor	With the factor	
Diabetes	*n*	357	54	0.281	108	9	0.274
	*Age*	65.1 (7.7)	66.1 (7.6)		64.4 (7.9)	63.6 (9.2)	
	*Schooling*	11 (5–17)	8.5 (5–16)		11 (5–16)	14 (8–16)	
	*MoCA*	26 (24–28)	26 (24–28)		26 (24–28)	27 (26–28)	
High blood pressure	*n*	244	167	0.009	72	45	0.777
	*Age*	64.3 (7.5)	66.6 (7.7)		62.8 (7.4)	66.9 (8.2)	
	*Schooling*	11 (5.5–17)	11 (5–17)		13 (7–17)	8 (5–12)	
	*MoCA*	26 (25–28)	26 (23–27)		26 (24–28)	26 (24–28)	
Overweight	*n*	181	230	0.120	47	70	0.862
	*Age*	65.4 (8.2)	65.1 (7.3)		64.8 (7.1)	64.1 (8.5)	
	*Schooling*	13 (8–17)	11 (5–16)		12 (7–17)	10 (5–14)	
	*MoCA*	26 (24–28)	26 (24–27)		26 (24–28)	26 (24–27)	
Dyslipidemia	*n*	296	115	0.998	65	52	0.835
	*Age*	65.3 (7.8)	65.1 (7.4)		64.4 (8.9)	64.4 (6.7)	
	*Schooling*	11 (5–17)	11 (5–17)		11 (6–16)	10 (5–16)	
	*MoCA*	26 (24–28)	26 (24–27)		26 (25–27)	26 (24–28)	
Schooling≤5 years	*n*	296	115	0.000	85	32	0.010
	*Age*	64.5 (7.7)	67.1 (7.4)		63.9 (7.5)	65.5 (9.2)	
	*Schooling*	15 (11–17)	5 (2–5)		13 (11–17)	5 (3–5)	
	*MoCA*	27 (25–28)	24 (20–26)*		26 (25–28)	24.5 (21.5–27)*	
Schooling≤10 years	*n*	241	170	0.000	64	53	0.048
	*Age*	64.1 (7.7)	66.8 (7.3)		62.6 (7.7)	66.5 (7.7)	
	*Schooling*	16 (12–17)	5 (3–7)		15 (12.5–17)	5 (4–7)	
	*MoCA*	27 (25–28)	25 (22–27)*		26 (25–28)	25 (22–28)*	
Age≥70 years old	*n*	293	118	0.043	94	23	0.007
	*Age*	61.3 (4.7)	74.9 (4.2)		61.4 (5.4)	76.4 (4.5)	
	*Schooling*	11 (6–17)	9.5 (5–16)		11 (6417)	8 (4–12)	
	*MoCA*	26 (25–28)	26 (23–27)*		26 (25–28)	24 (22–26)*	

* Statistically significant difference in MoCA test between the group with risk factor and the group without the risk factor, according to the Mann-Whitney U test (p<0.05).

### Subjects with MCI: MoCA-test performance in ϵ4 carriers and noncarriers and their different risk factors

In subjects with MCI, the results were similar to those observed in individuals with normal cognition; a lower MoCA-test score was found in those who had equal or less than five years of schooling compared to subjects with more than 5 years of schooling also in both ϵ4 allele carriers and noncarriers. In the MoCA-test for a schooling of five years or less, carriers: median: 19 [IQR: 16–21] *versus* 22 [IQR: 20–23.5], p*=*0.000, Cliff’s delta=-0.50 (large); noncarriers: median 20 [IQR: 16–22]) *versus* 21 [IQR: 19–23], p*=*0.000, Cliff’s delta=-0.29 (small). The same was observed in subjects with equal or less than 10 years of schooling compared to those who had 11 or more years of schooling, carriers: median: 20 [IQR: 18–22] *versus* 23 [IQR: 22–24], p*=*0.000, Cliff’s delta=0.52 (large); noncarriers: median 20 [IQR: 17–22]) *versus* 22 [IQR: 19–24], p*=*0.000, Cliff’s delta=0.32 (small).

Similar results were observed in subjects of 70 years of age or older who had a lower MoCA-test score compared to younger elderly population, carriers: median: 20 [IQR: 18–22] *versus* 23 [IQR: 22–24], p*=*0.011, Cliff’s delta=0.30 (small); noncarriers: median: 20 [IQR: 17–22]) *versus* 21 [IQR: 19–23], p*=*0.000, Cliff’s delta=0.23 (small).

However, a lower score in MoCA test was observed in subjects with high blood pressure, noncarriers with the factor compared to noncarriers without factor (although the median was similar, the Mann-Whitney U test detected differences: median: 20 [IQR: 17–22] *versus* 20 [IQR: 18–23], p*=*0.022, Cliff’s delta=0.14 (negligible).

For the other risk factors, being a ϵ4 allele carrier or not and being diabetic, overweight, or having dyslipidemia was not related to the performance in the MoCA-test, in subjects with MCI (Table 3 and Figure 1).

**Table 3.** Comparison of Montréal Cognitive Assessment Test, by risk factors, between participants with mild cognitive impairment, carriers and non-carriers of the allele ε4.

Risk factor		Non-ε4 allele carrier		p-value	ε4 allele carrier		p-value
		Without the factor	With the factor		Without the factor	With the factor	
Diabetes	*n*	278	45	0.141	77	14	0.319
	*Age*	69.3 (7.8)	69.1 (8.9)		68.9 (7.0)	68.3 (7.5)	
	*Schooling*	5 (3–11)	5 (3–11)		7 (3–11)	8.5 (5–12)	
	*MoCA*	20 (18–22)	19 (17–22)		22 (19–23)	20.5 (19–23)	
High blood pressure	*n*	165	158	*0.022*	54	37	0.437
	*Age*	68.4 (8.7)	70.3 (7.2)		68.3 (6.5)	69.6 (7.8)	
	*Schooling*	6 (4–11)	5 (3–11)		7.5 (5–11)	7 (3–11)	
	*MoCA*	20 (18–23)	20 (17–22)*		22 (19–23)	21 (18–23)	
Overweight	*n*	130	193	0.639	47	44	0.869
	*Age*	70.6 (8.5)	68.4 (7.6)		69.5 (7.1)	68.0 (7.0)	
	*Schooling*	5.5 (3–10)	5 (3–11)		8 (3–12)	6.5 (3–11)	
	*MoCA*	20 (17–22)	20 (18–22)		22 (18–23)	21 (19–23)	
Dyslipidemia	*n*	208	115	0.179	53	38	0.150
	*Age*	69.1 (8.3)	69.6 (7.5)		68.5 (7.1)	69.3 (7.0)	
	*Schooling*	5 (4–11)	5 (3–11)		8 (3–11)	6.5 (3–12)	
	*MoCA*	20.5 (18–22)	20 (17–22)		20 (18–23)	22 (20–23)	
Schooling<=5 years	*n*	151	172	0.000	52	39	0.000
	*Age*	68.3 (8.5)	70.1 (7.5)		66.7 (6.5)	71.6 (6.8)	
	*Schooling*	11 (8–14)	3 (2–5)		11 (8–15.5)	3 (2–5)	
	*MoCA*	21 (19–23)	20 (16–22)*		22 (20–23.5)	19 (16–21)*	
Schooling≤10 years	*n*	89	234	0.000	32	59	0.000
	*Age*	66.8 (8.7)	70.2 (7.5)		66.8 (6.9)	69.9 (6.9)	
	*Schooling*	13 (11–17)	5 (3–6)		12.5 (11–17)	5 (2–7)	
	*MoCA*	22 (19–24)	20 (17–22)*		23 (22–24)	20 (18–22)*	
Age≥70 years old	*n*	155	168	0.000	51	40	0.011
	*Age*	62.5 (4.6)	75.6 (4.7)		63.8 (4.2)	75.2 (4.4)	
	*Schooling*	5 (3–11)	5 (3–9.5)		9 (5–13)	5 (2–8)	
	*MoCA*	21 (19–23)	19 (17–22)*		22 (20–23)	20 (17–22)*	

* Statistically significant difference in MoCA test between the group with risk factor and the group without the risk factor, according to the Mann-Whitney U test (p<0.05).

### Subjects with dementia: MoCA-test performance in ϵ4 carriers and noncarriers and their different risk factors

For patients with dementia, a lower MoCA-test score was observed, regardless if they were ϵ4 allele carriers or not in those who had HBP compared with subjects without this risk factor, carriers: median 13 [IQR: 12–15] *versus* 15 [IQR: 14–17], p*=*0.043, Cliff’s delta=0.30 (small); noncarriers: median 12.5 [IQR: 9.5–16] *versus* 14 [IQR: 12–16], p*=*0.032, Cliff’s delta=0.30 (small) and in whom schooling was less than 5 years, carriers: median 14 [IQR: 12–15] *versus* 16.5 [IQR: 14.5–18.5], p*=*0.000, Cliff’s delta=-0.57 (large); non-carriers: median: 13 [IQR: 10–15] *versus* 15 [IQR: 13–17], p*=*0.000, Cliff’s delta=-0.38 (medium).

Although, there was a tendency to lower scores in diabetic ϵ4 carriers compared with noncarriers, carriers: median 13 [IQR: 9–14] *versus* noncarriers 14 [IQR: 13–16]); however, this was not statistically significant (p*=*0.066).

In the same way, no statistically significant differences were observed between subjects with and without the other risk factors, both carriers and noncarriers (Table 4).

**Table 4.** Comparison of Montréal Cognitive Assessment test, by risk factors, between patients with dementia carriers and non-carriers of the allele ε4.

Risk factor		Non-ε4 allele carrier		p-value	ε4 allele carrier		p-value
		Without the factor	With the factor		Without the factor	With the factor	
Diabetes	*n*	178	35	0.215	49	12	0.066
	*Age*	73.3 (8.9)	73.7 (8.4)		70.6 (7.5)	75.5 (7.9)	
	*Schooling*	3 (2–5)	4 (1–5)		4.5 (2–6)	2 (0–5)	
	*MoCA*	13 (11–16)	12 (9–15)		14 (13–16)	13 (9–14)* 0.06	
High blood pressure	*n*	85	128	0.032	24	35	0.043
	*Age*	71.7 (8.6)	74.4 (8.8)		70.6 (8.3)	71.8(7.6)	
	*Schooling*	4 (2–5)	3 (1–5)		5 (2.5–8.5)	4 (1–5)	
	*MoCA*	14 (12–16)	12.5 (9.5–16)*		15 (14–17)	13 (12–15)*	
Overweight	*n*	76	137	0.038	25	34	0.111
	*Age*	75 (8.9)	72.4 (8.6)		71.7 (8.6)	71.1 (7.3)	
	*Schooling*	4 (2–5)	3 (1–5)		5 (3–9)	3 (1–5)	
	*MoCA*	12 (9.5–15)	13 (11–16)*		15 (14–16)	13.5 (12–16)	
Dyslipidemia	*n*	154	61	0.308	34	25	0.694
	*Age*	73.9 (8.7)	71.8 (8.8)		72.9 (7.7)	69.1 (7.6)	
	*Schooling*	4 (1.5–5)	3 (2–5)		4 (2–6)	4 (2–5)	
	*MoCA*	13 (10–16)	13 (11–16)		14 (12–16)	14 (12–16)	
Schooling≤5 years	*n*	39	174	0.000	16	43	0.000
	*Age*	70.5 (10.3)	73.9 (8.3)		72.3 (6)	70.9 (8.4)	
	*Schooling*	9 (7–13)	3 (1–5)		9 (8–11)	3 (1–5)	
	*MoCA*	15 (13–17)	13 (10–15) *		16.5 (14.5–18.5)	14 (12–15)*	
Schooling≤10 years	*n*	19	194	0.000	6	53	0.106
	*Age*	71.8 (10.2)	73.5 (8.6)		72.8 (2.8)	71.3 (8.1)	
	*Schooling*	13 (11–17)	3 (1–5)		11 (11–12)	3 (2–5)	
	*MoCA*	16 (14–17)	13 (10–15)*		16 (14–19)	14 (12–16)	
Age≥70 years old	*n*	73	140	0.000	26	33	0.345
	*Age*	63.5 (4.3)	78.5 (5.6)		64.3 (3.3)	76.8 (5.6)	
	*Schooling*	4 (2–7)	3 (1–5)		3 (2–5)	5 (2–8)	
	*MoCA*	14 (12–16)	12.5 (9–15)*		14 (13–16)	14 (12–16)	

* Statistically significant difference in MoCA test between the group with risk factor and the group without the risk factor, according to the Mann-Whitney U test (p<0.05).

**Figure 2.**
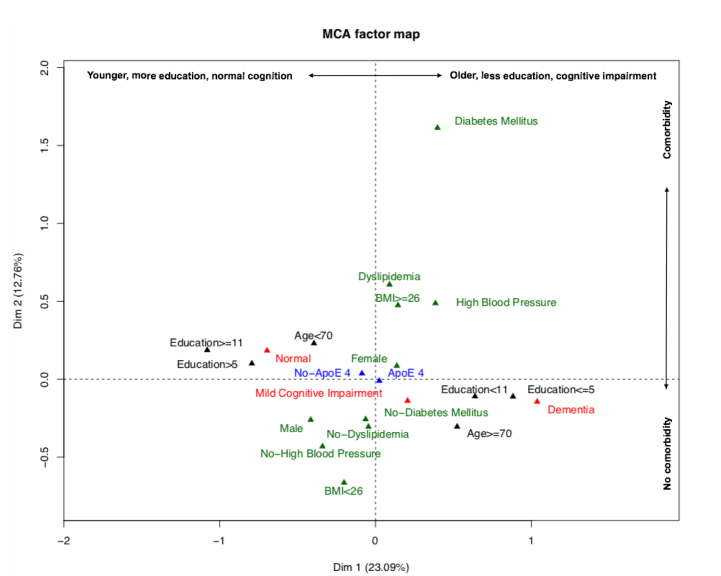
Factorial plane representing the relationship between risk factors, cognitive impairment, and condition of the allele ε4.

The multiple correspondence analyses for patients with cognitive impairment presented an adjusted inertia for the first four dimensions of 23.09, 12.76, 11.60, and 10.45%, respectively. The first dimension explains 23.09% of the variability of the data and the categories are organized mainly along this axis. The best representation of the variables was achieved in the first two dimensions, reaching 35.84% inertia (Figure 2).

BMI: body mass index. The relationship between these variables is explained in two dimensions: vertical axis includes schooling, age, and cognitive status; horizontal axis includes cardiovascular factors.

Figure 2 shows that the presence or absence of ϵ4 allele, does not discriminate special groups, as the two categories (ϵ4 carrier vs non-carrier) are very close to the origin, which is consistent with the previous analyzes; being an ϵ4 carrier is not associated with the other variables considered, although special relationships are observed on the axes: on axis 1 the educational level, degree of cognitive impairment and age are represented; axis 2 represents the presence or absence of comorbidities such as dyslipidemia, HBP, DM and overweight.

### Analysis of the interaction between being a ϵ4 allele carrier and risk factors

After adjusting age, when evaluating the interaction between being ϵ4 allele carrier and having some risk factors, it was observed that the decrease in the MoCA-test score in the three groups of patients (normal, MCI, and dementia) was related to low schooling (≤ 5 years or ≤10 years), regardless of the status of the ϵ4 allele.

In addition, in patients with MCI a decrease of one point in the median MoCA test score was observed, in carrier subjects with high blood pressure *versus* noncarriers without high blood pressure.

On the basis of the results described above, patients with dementia carrying ϵ4 allele and without overweight showed a decrease of 2.58 points in the median of the MoCA-test score. In all comparisons that reached statistical significance, the greatest decreases in the MoCA-test score were observed in subjects with dementia (Table 5 and Figure 2).

**Table 5.** Relationship between the Montréal Cognitive Assessment test and the age-adjusted interaction between risk factors and allelo ε4.

Factor	Normal		Mild cognitive impairment		Dementia	
	β	p-value	β	p-value	β	p-value
Diabetes						
Non-ε4 allele carrier, no diabetes	Ref.		Ref.		Ref.	
Non-ε4 allele carrier, with diabetes	−0.40	0.337	−0.82	0.237	−0.66	0.455
ε4 allele carrier, no diabetes	−0.40	0.202	0.87	0.119	1	0.203
ε4 allele carrier, with diabetes	0.67	0.489	0.04	0.967	−0.53	0.734
Hypertension						
Non-ε4 allele carrier, no hypertension	Ref.		Ref.		Ref.	
Non-ε4 allele carrier, with hypertension	−0.52	0.074	−1	0.046	−1.12	0.043
ε4 allele carrier, no hypertension	−0.58	0.135	0.33	0.633	1.56	0.086
ε4 allele carrier, with hypertension	−0.58	0.217	−0.00	1.000	0	1.000
Overweight						
Non-ε4 allele carrier, unweight	Ref.		Ref.		Ref.	
Non-ε4 allele carrier, overweight	−0.50	0.091	−0.09	0.858	0.76	0.224
ε4 allele carrier, unweight	−0.81	0.096	1.00	0.186	2.58	0.011
ε4 allele carrier, overweight	−0.43	0.297	0.63	0.413	1	0.270
Dyslipidemia						
Non-ε4 allele carrier, no dyslipidemia	Ref.		Ref.		Ref.	
Non-ε4 allele carrier, with dyslipidemia	−0.14	0.659	−0.63	0.205	0	1.000
ε4 allele carrier, no dyslipidemia	−0.21	0.595	−0.27	0.681	1	0.281
ε4 allele carrier, with dyslipidemia	−0.78	0.076	1.27	0.095	1	0.349
Schooling ≤5 years						
Non-ε4 allele carrier, >5 years	Ref.		Ref.		Ref.	
Non-ε4 allele carrier, ≤5 years	−2.41	0.000	−1.70	0.000	−2.52	0.001
ε4 allele carrier, >5 years	−0.52	0.166	0.80	0.204	0.76	0.550
ε4 allele carrier, ≤5 years	−2.41	0.000	−2.1	0.003	−1.61	0.089
Schooling ≤10 years						
Non-ε4 allele carrier, >10 years	Ref.		Ref.		Ref.	
Non-ε4 allele carrier, ≤10 years	−1.93	0.000	−2	0.000	−3	0.003
ε4 allele carrier, >10 years	−0.75	0.128	0.72	0.407	0.71	0.971
ε4 allele carrier, ≤10 years	−1.68	0.002	−2.09	0.004	−2	0.078
Age≥70 years old						
Non-ε4 allele carrier, <70 years	Ref.		Ref.		Ref.	
Non-ε4 allele carrier, ≥70 years	0.42	0.377	0.09	0.912	−2	0.003
ε4 allele carrier, <70 years old	−0.07	0.826	1.09	0.143	0.00	1.000
ε4 allele carrier, ≥70 years old	−1.21	0.106	0.18	0.860	0.00	1.000

## DISCUSSION

Being an ϵ4 carrier has been recognized as the main genetic risk factor associated with the development of late-onset AD.^8^ The percentage distribution of the isoforms described in the literature for the alleles of the APOΕ gene is 79% for the ϵ3 allele, 13.3% for the ϵ4 allele, and 7.3% for the ϵ2 allele.^27^ ^,^ ^28^ The genotype frequencies of the APOΕ gene in our sample studied were similar to those described in Colombian, Latin American, and world literature.^26^ A hazard ratio (HR) of 1.35 (95%CI 1.00–1.83) has been reported between the presence of the ϵ4 allele and the risk of AD,^29^ whereas in a previous study in Colombia, it has been observed that the relationship has an OR of 5.1.^30^

A relationship between age, sex, Apoϵ4, and cognitive impairment has been described.^11^ In this study, regardless of gender and being ϵ4 carrier, those subjects over 70 years have a higher risk of cognitive impairment.

We also found a significant association between low schooling (≤5 years)and a lower performance in the MoCA-test, regardless if they were or not ϵ4 allele carriers and their cognitive status, results that are concordant with Borland et al., who found that low schooling (≤10 years) was associated with significantly lower MoCA-test scores and concluded that schooling is a significant predictor for the MoCA-test score.^31^ ^,^ ^32^ Similar results were described by Conti et al., who after analyzing 225 healthy subjects with 5 or less years of schooling, observed that it influenced the final score of the test, especially in those subjects with only 1 year of schooling (p<0.0001).^33^

Accordingly, the difference between MoCA-test scores among subjects with low and high schooling in our study on average was 1.5 points, data that are consistent with those reported by Konstantopoulos et al.,^34^ who find an average difference of 1.4 points comparing MoCA-test results for 1–9 years of schooling (low schooling), 10–12 years of schooling (high school) and above 13 years (bachelor or higher education).

In other studies, the interaction among cognitive impairment, Apoϵ4, and schooling presents contradictory evidence; Seeman et al.,^35^ Ishioka,^36^ and Vemuri et al.^37^ observed that the presence of at least one ϵ4 allele reduces the protective effect of education on cognitive function, evidenced by a lower score of Short Portable Mental Status Questionnaire in subjects carrying ϵ4 and with at least 9 years of schooling. On the contrary, Sando et al. found that a higher educational level was related to a lower OR (participants with 10–18 years of education showed a lower OR purchased with subjects with 6–7 years of education); however, the protector effect of education on the onset of dementia wasn’t modified by the presence of ϵ4 alleles;^38^ These studies are in agreement with our results and with those of Sánchez et al., who failed to demonstrate a relationship between educational level, cognitive impairment (MCI or dementia) and being ϵ4 carrier.^39^ Recent studies confirm the variability of this interaction.^40^

Other studies have described the relationship between the presence of the ϵ4 allele, some cardio-metabolic factors, and the development of cognitive impairment, which suggests that the presence of this allele would modify the association between dementia and HBP.^41^ ^‒^ ^42^ In our study we found that subjects with HBP had a lower performance in the MoCA test, regardless of whether they were ϵ4 allele carriers or not; similar studies have been performed using the Mini-mental test (MMSE) and the MoCA test.^43^ ^,^ ^44^ ^,^ ^45^

When assessing whether the scores obtained in the MoCA test in subjects ϵ4 carriers with HBP were different from those being HBP non-ϵ4 carriers, our study found no differences. When analyzing the independent relationship of the MoCA test scores, Bangen et al.^14^ found that vascular risk factors, such as HBP, provide a higher risk of cognitive impairment and could be strengthened by the presence of the ϵ4 allele.^25^ In addition, Weinstein et al. analyzed the relationship between ϵ4 allele and HBP (measured within the Framingham scale and changes in the MoCA-test score); they found that high scores on this scale of cardiovascular risk factors were related to low performance on the cognitive scale, this relationship wasn’t modified by the presence of the ϵ4 allele, as we found in our study.^46^

On the contrary, our study showed a lower performance of the MoCA-test in subjects with dementia, ϵ4 carriers diabetics compared to ϵ4 carriers non-diabetic. The relationship among AD, APOϵ4, and DM was assessed by Haan et al., who described that the ϵ4/ϵ4 genotype contributes synergistically with type 2 diabetes (DM2) in the development of AD.^47^ Similar data were described by Zhao et al.^48^ and Johnson et al.,^49^ who reported that ϵ4 allele carriers diabetic had a higher prevalence of AD compared with noncarriers ϵ4 diabetics subjects (7.55 vs. 2.3%), with a risk probability of 3,982 [95%CI 1,418–11,184] of developing dementia in diabetic subjects and ϵ4 carriers, however, additional studies are still required to demonstrate this relationship, as stated by Shinohara M et al in the most recent review of 2020.^50^

Differences in cognitive scales between diabetic carriers and noncarriers were described by Palmer et al., who reported that ϵ4 diabetic patients had lower scores on the MMSE test compared to noncarriers and non-diabetic subjects.^51^ On the contrary, Zhen et al. showed that subjects with at least one ϵ4 allele and diabetics obtained lower scores on the MoCA test, independently of cognitive status.^52^

The presence of risk factors such as overweight or dyslipidemia were not related to the performance in the MoCA test, in ϵ4 allele carriers and noncarriers. The relationship of overweight, poor performance on cognitive tests, and the presence of ϵ4 allele have been poorly documented and its results are contradictory. In this regard, Blautzik et al.^53^ observed that on ϵ4 carriers, the BMI had an inverse relationship with cognitive impairment (β=-0.209, p=0.05), higher BMI showed lower scores on cognitive tests;^54^ Recent studies of this same group of subjects showed similar results to those of Blautzik et al.,^53^ where the subjects who acquired dementia over time, had normal or low BMI suggesting that low weight was a more important risk factor than overweight.^55^ ^,^ ^56^ ^,^ ^57^

Regarding the relationship among cognition, dyslipidemia, and APOϵ4, Reitz et al., Hayden et al.,^59^ and Wei et al.^60^ described an association among lipid metabolism, AD, and ϵ4 allele, but the mechanisms still require further research and larger sample sizes.^58^ ^‒^ ^60^

Study limitations: ϵ4 carrier population was smaller than the non-ϵ4 carrier in most of the different risk factor groups, a fact that could have interfered in the association forces between the different risk factors and the presence of ϵ4 allele.

We found that subjects with low schooling (≤5 years or ≤10 years) and subjects with HBP had lower performances in the MoCA-test scores, regardless of being ϵ4 carrier or not, and presenting or not cognitive impairment.

Being an ϵ4 carrier and diabetic showed a tendency to present the lowest scores in the MoCA-test only in subjects with dementia as compared to diabetic subjects with dementia noncarriers ϵ4.

A future study with a larger cohort and a longer longitudinal follow-up time could show us a greater effect of apoϵ4 with risk factors on cognitive decline.

Extensive continuing education programs, in all age groups of our society, with a better control of risk factors and promotion of healthy lifestyles, are the best options currently available to reduce the onset and progression of cognitive decline.
